# Essays on the Uses and Properties of Some Remedies

**Published:** 1830-05

**Authors:** John Gardner

**Affiliations:** Surgeon, &c.


DULCAMARA.

### *Essays on the Uses and Properties of some Remedies.* By John Gardner, Surgeon, &c.

### 1. DULCAMARA.

*Preliminary Observations.* The sciences of morbid anatomy and pathology are doubtless of infinite importance to the art of curing diseases; but the present prevailing taste disposes the student to give these subjects his attention too exclusively, and to overlook another object of inquiry of not less importance or interest, namely, the precise effects of remedies upon the body in disease. After carefully looking through the Dispensatories, and other works on Materia Medica and Therapeutics, as well as the most approved authors on Medicine, the conclusions are warranted that there is a great deficiency of this species of information, that the phenomena of the influences of drugs have not been minutely enough observed, their analysis ascertained, or their peculiarities discriminated. We are disposed, however, to attribute this deficiency rather to the literature of medicine, than to absolute ignorance on the part of medical men. There can be no doubt that much of the advantage experience gives to the older practitioner depends on his knowledge of the minute circumstances of the operation of remedies, and that considerations, not necessary to be discussed here, prevent him from communicating that information to the world.

The inquiries most interesting to myself are, what is the remedy for a certain symptom or train of symptoms? what the precise influence of a remedy? A large majority of cases met with in private practice call upon us to correct a disturbed function, to relieve a painful sensation, or to check a diseased action, of a kind and degree that we can confidently pronounce will not lead to fatal changes of structure, even if neglected; that nature, unassisted, will effect a cure, however slowly. But to relieve pain, and to restore the sufferer to his wonted occupations and pleasure, we must have recourse to means of cure the most rapid and effectual we possess. There are many diseases too certainly irresistibly fatal, which leave no change of structure in the body, as far as we have yet discovered; and there are also many changes of structure obvious enough, because seen in their whole progress, as in diseases of the skin and superficial glands, which are by no means on that account easier to cure.

We would not be supposed by these remarks to intend any disparagement of the study of morbid anatomy; our desire is to show that the inquiry here recommended is fully equal in importance, and not less so in interest.

In attempting to ascertain distinctly the powers of remedies, we have many and formidable difficulties to overcome, the explanation and elucidation of which would require a volume; but if we are animated by a desire to elicit truth, rather than to establish preconceived hypotheses or hasty generalizations, these difficulties will quickly yield, and we cannot fail to be rewarded by important results: for, after all that has been done for the art of healing in ancient or modern times, this presents the most promising field for discoveries, especially to those who possess industry and zeal for their profession, more than commanding talents or genius.

The progress of the study of natural history and botany enables us to discriminate any particular object in the whole range of organized beings, and the judicious application of chemical research to them has enabled us to separate the power, as it were, of each substance from the inert materials with which in nature it is combined. When we are thus furnished with remedies in the most useful forms, we cannot know their effects on the animal body before actual experiment has been made; and when we know those effects on the healthy body, it teaches us nothing of their influences on disease: this remains a distinct subject of investigation. Some substances, absolutely inert during health, exercise remarkable powers over disease; and others, highly deleterious to the healthy body, may be administered with impunity, or even with great benefit, in many diseases. The failure of remedies to produce the same effects on diseases under every variety of circumstances, has often led to their neglect and total disuse. In some instances this may have arisen from the use of a different substance to that intended, in consequence of adulteration or the fraudulent substitution of another for it. But when every care has been taken to use the unadulterated material, the want of effect often disappoints us; the exact state of disease for which alone it is the remedy not being present. Close analogy having been mistaken for identity, the group of associated symptoms determining the place of a disease in our systems of nosology, and to which a name is given, is seldom complete or unmodified; it never indicates the constitutional state of the individual diseased, and is moreover frequently selected for reasons altogether hypothetical. Hence it has happened that nosological arrangements have retarded that minute knowledge of symptomatology required in studying the effects of remedies: and, again, the habit of generalizing, so important in other sciences, has, in this particular department, been an instrument of error; for when a variety of medicines have been observed to agree in one or two particulars of their action on the animal economy, they are arranged into classes, and we have sedatives, diuretics, cathartics, &c. The several remedies of each class have too often been employed indiscriminately, and for the purpose of producing the effect common to the whole. In arranging medicinal agents into classes, and in studying their *modus operandi,* more hypotheses have prevailed, and more deceptive analogies have been admitted, than in any other department of medical knowledge. To illustrate the unphilosophical process of induction common in inquiries on this subject, we may relate of a celebrated teacher and author, that, in his public lectures, he condemns, in unmeasured terms, as guilty of no small crime, all those who administer medicines without knowing the principle on which they act. What he means by the *principle* on which medicines act, we learn from his own examples in explanation: Alkalies are useful in some forms of dyspepsia; the principle of this is to be explained, and for this purpose two hypotheses are had recourse to. Dyspepsia, says he, is a gastric irritation; alkalies allay this irritation; they are therefore sedatives, and must be classed as such. The error, or at least the utter inutility of this mode of reasoning, is obvious. Such principles may mislead the student, who, in the first case of dyspepsia presented to his notice, would employ alkalies: should they fail, he might be induced to abandon their use in dyspepsia altogether. In truth, every practitioner knows that there are some disturbances of the stomach, some forms of dyspepsia, for which alkalies are almost certain remedies. Instead, therefore, of merging all disturbances of the stomach into an irritation, a gastric embarrassment, or any other general term, we should endeavour to ascertain by the symptoms, minutely and accurately observed, the varieties and differences of the several kinds of these disturbances, carefully noting the symptom or symptoms pathognomic of the state of the structure, but more particularly regarding the symptom or combination of symptoms indicative of the agent that will cure the disease. This indication will, of course, depend on experience, obtained by minute observation and experiment; not made by trying a farrago of heterogeneous materials, without order or reason, but conducted on strictly philosophical principles.

In the study of remedies, it appears that, instead of paying exclusive attention to the properties in which they agree, we should endeavour to ascertain in what respect apparently similar substances differ in their action on the animal economy under disease. As, in language, words nearly synonymous have really differences of meaning, and although in some cases they may be used indiscriminately, yet in others one cannot be substituted for another; so with respect to substances employed as remedies, each produces some peculiar effect, essentially different to the effect of every other; and the same may be asserted of every article of food, and of those subtle agents which modify the character of the air we breathe. These peculiar effects we may be permitted to denominate the specific effects of remedies, to distinguish them from those effects produced by them in common with a class to which they may belong. Thus, the influence of belladonna on the iris is its specific effect, and, as far as its use as a medical agent is concerned, is essentially different to the power of inducing sleep common to all narcotics. The specific effects of the more powerful agents are better known, because they are more quickly manifested. Of those less powerful, it often is necessary that they should be exhibited for a long period. We are not always desirous of inducing these specific effects; for almost every agent manifests two classes of effects, immediate and remote; the former usually ensured by a large dose, the latter by a long continuance of smaller doses: either of these may be the specific effect of the remedy. It is a point of no small importance in the study of the *modus operandi* of medicines, to ascertain when it is necessary that the specific effect should be produced, in order to cure any disease. In chronic diseases we believe this generally to be required. We may illustrate this by a reference to mercury: in syphilis, and some other diseases, its specific effect on the salivary glands is often necessary before the disease can be cured; in other cases we carefully avoid pushing the remedy to this result. In the case of other remedies employed in the cure of chronic diseases, we are not justified in determining their want of influence on the disease until some effect is produced on some part of the constitution, the *specific* effect of the remedy; and this will serve as an indication that the remedy has been fairly tried. We are acquainted with the true specific effects of very few substances; but that these are usually the most important *remedial* effects, will appear manifest when we consider what those are on which we rely with most confidence. Sulphur, mercury, bark, colchicum, iodine, ergot, &c.: to what general principle can we refer the peculiar influence any one of these exerts over certain diseases? We seldom, if ever, employ them for the effects they produce in common with other remedies: for example, the use of colchicum as a diuretic was long since abandoned, yet how valuable is it in gout and rheumatism. We believe, however, that, had attention been directed to the minute circumstances attendant on those diseases, and the phenomena of the action of the remedy in other cases, their adaptation might have been much earlier known. Now, there are numberless agents possessing strong claims to our attention, the specific effects of which are altogether unknown: some which have been used formerly with great repute, and now abandoned, and others in daily use for their common and more obvious properties. This may serve in some measure to explain the extraordinary discrepancy of opinion among practitioners on the efficacy of certain remedies. Who that practises medicine does not know that there are many diseases, many painful sensations, and morbid actions, over which we have at present little or no control? yet we may justly be allowed to expect (if the foregoing remarks are well founded,) that, by well-directed research, our remedial powers will be indefinitely increased.

The principles briefly glanced at in the preceding observations are perhaps worthy of being more systematically developed and illustrated. In what follows on the subject of the properties of dulcamara, we have given an example of the method of studying the effects of remedies which is recommended, and in that example shown to what kind of results it is likely to lead.

*On Dulcamara.*

In 1828, I was consulted by an old lady, aged seventy-two, concerning a disease of the skin, from which she had suffered for nine months. She had no hope of being cured, but was desirous of obtaining some temporary relief. It would be impossible to assign a place in nosology to the disease. It was scaly, not in defined circumscribed patches, but continuous, with a deep red blush on the cutis beneath; an ichor constantly oozed from the surface; small vesicles might here and there be observed; frequently a crop of minute pustules, like those of tinea capitis; and sometimes large vesicles would rise after burning pain, simulating erysipelas. This horrible eruption, in one or other of these forms, covered the whole body of the patient. The smarting and itching were constant, and almost intolerable, and the burning pain recurred frequently: it was worst on the arms, legs, and thighs. The poor woman's health was, of course, much affected; she was reduced to a mere skeleton; she had totally lost her appetite, had continued thirst, and constipated bowels; was usually so chilly as to be obliged to keep constantly near a large fire, even in summer, and seldom enjoyed an hour's sleep at night. She had been under the care of several medical men, and had tried mercury in every variety of form and degree, externally and internally; acids, bark, and other tonics; arsenic, alkalies, opium, &c.; from none of which had she experienced any relief. For some time, however, she had merely employed a liniment of oil, opium, and subacetate of lead, to sooth the irritation, and various purgatives, to obviate habitual costiveness.

I first tried the Conium, externally and internally, until it manifested its effects on the system, but without the slightest benefit to the disease. If any remedy would relieve the symptoms by its narcotic quality, why should not conium or opium? The Solanum Dulcamara is frequently used in skin diseases: it is classed as a narcotic, but I could find no satisfactory estimate of its powers. Mr. Brande, in his Manual of Pharmacy, says of it, "The decoction of these stalks, (the Solanum Dulcamara,) which should be gathered in autumn, has a bitterish sweet taste, and operates both as a narcotic and diuretic. In very large doses, it produces the usual symptoms of the narcotic poisons. I say nothing of its exhibition and doses, as it is a very uncertain and useless remedy. It has chiefly gained celebrity as an article in diet drinks, in certain cutaneous affections, and has been recommended as efficient in the relief of chronic rheumatism; but it is entitled to no manner of confidence either in these or other complaints." Thomson says, "In large doses it produces nausea, vomiting, syncope, and palpitation. If these symptoms occur, the dose must be lessened." He does not apparently understand its properties.

Notwithstanding these unfavorable testimonies, I determined on trying it, and, carefully procuring a good specimen of the dried herb, had a decoction prepared of twice the usual strength. It was given in doses of an ounce three times a day, with a little tincture of Cardamoms, and soon produced the effects so much dreaded, namely sickness and vertigo: nevertheless, it was persisted in, and in a few days a beneficial change was taking place. The patient no longer required purgatives, as the bowels were gently moved; the appetite improved, the irritation of the skin subsided, the eruption gradually became less, and the patient was in about a month restored to perfect health. The urine was increased in quantity while the medicine was taken, and these were all the sensible effects I could discover.

The next case in which I tried the Dulcamara, was that of a young lady, seven years of age. I was summoned to her at midnight, and found her in a state resembling apoplexy. She had for two years suffered from an eruption of an irritable character extending over the body, with ichorous oozing behind the ears, and small pustules frequently appearing on the scalp. A surgeon of high repute had prescribed an astringent ointment, which had rapidly stopped the discharge, and the symptoms I saw speedily followed. The usual remedies, bleeding, leeches, warm baths, blisters, &c. removed the alarming symptoms, and the eruption was restored to the skin. The Dulcamara, given in this case in the form of a syrup made with the extract, produced the same effects as in the former instance, namely, sickness, vertigo, and gentle catharsis. The eruption was speedily and effectually cured.

These cases suggested to me that it is possible the symptoms noticed by authors as subjects of dread, namely, the sickness, vertigo, &c., produced by the Dulcamara, might be the index of its full influence upon the system; the mark of its specific effect upon the disease. I have since had abundant opportunities of establishing the truth of this conclusion, and I can now confidently assert that, if properly administered, the Solanum Dulcamara is a most effectual remedy in skin diseases, especially those attended with irritation, pustules, vesicles, scales, &c. The diseases in which I have employed it with uniform success are psoriasis, in several varieties, impetigo, eczema, and porrigo, as well as in lepra and ichthyosis. In a great number of obstinate cases of porrigo it has succeeded without any local application.

To ensure success from the use of Dulcamara, it is necessary that it should be collected at a proper time, and carefully dried; it should, when dry, be capable of yielding a powder of a bright green colour, and, by watery infusion, an extract possessing strongly the peculiar flavor and odour of the fresh plant. With these precautions, it may be given in the forms of powder, decoction, infusion, pills, or syrup. Perhaps it is best always to use the extract, which contains concentrated the peculiar properties of the plant; but it must always be began in small doses, and gradually increased until sickness, vertigo, and purging are produced. For some constitutions are peculiarly susceptible to its influence, whilst others resist it until very large doses have been given. In no case have I known any benefit to the skin disease from its use, unless the symptoms of its influence on the system were produced. It is necessary, of course, when these symptoms occur, to proceed with caution, to properly diminish the dose, or lengthen the intervals of exhibiting it, in the same manner and for the same reasons as we should do with digitalis, or any other powerful remedy.

I may, in conclusion, remark that several cases have occurred to me where the patient has taken, by the advice of physicians, the decoction of Dulcamara of the Pharmacopœia, prepared by some celebrated chemists, for a long period, without any effect. On inspecting the decoction, it has been a pale, dirty green, thin fluid, without much odour or taste. This slovenly mode of trying any remedy, may well lead to its disuse. The decoction ought to be of a dark bottle-green colour, depositing a copious sediment as it cools, which should be shaken into the fluid, and ought, at any rate, to be given until some effect is manifested.

The next essay will be on the medical history and properties of Lead; and, notwithstanding the attention this remedy has received of late, it will be found the subject has not by any means been exhausted. It is only by subjecting every remedy to a fair scrutiny that we can hope to give precision and accuracy to this most uncertain and unsatisfactory department of medical science.

---

